# Blurred lines: Performance Enhancement, Common Mental Disorders and Referral in the U.K. Athletic Population

**DOI:** 10.3389/fpsyg.2016.01067

**Published:** 2016-07-13

**Authors:** Claire-Marie Roberts, Andrea L. Faull, David Tod

**Affiliations:** ^1^Institute of Sport & Exercise Sciences, University of WorcesterWorcester, UK; ^2^School of Sport and Exercise Science, Liverpool John Moores UniversityLiverpool, UK

**Keywords:** common mental disorders, referral, elite athletes, case study, clinical psychology

## Abstract

Through the awareness-raising efforts of several high-profile current and former athletes, the issue of common mental disorders (CMD) in this population is gaining increasing attention from researchers and practitioners alike. Yet the prevalence is unclear and most likely, under-reported. Whilst the characteristics of the sporting environment may generate CMD within the athletic population, it also may exacerbate pre-existing conditions, and hence it is not surprising that sport psychology and sport science practitioners are anecdotally reporting increased incidences of athletes seeking support for CMD. In a population where there are many barriers to reporting and seeking help for CMD, due in part to the culture of the high performance sporting environment, anecdotal reports suggest that those athletes asking for help are approaching personnel who they are most comfortable talking to. In some cases, this may be a sport scientist, the sport psychologist or sport psychology consultant. Among personnel in the sporting domain, there is a perception that the sport psychologist or sport psychology consultant is best placed to assist athletes seeking assistance for CMD. However, sport psychology as a profession is split by two competing philosophical perspectives; one of which suggests that sport psychologists should work exclusively with athletes on performance enhancement, and the other views the athlete more holistically and accepts that their welfare may directly impact on their performance. To add further complication, the development of the profession of sport psychology varies widely between countries, meaning that practice in this field is not always clearly defined. This article examines case studies that illustrate the blurred lines in applied sport psychology practice, highlighting challenges with the process of referral in the U.K. athletic population. The article concludes with suggestions for ensuring the field of applied sport psychology is continually evolving and reconfiguring to ensure that it continues to meet the demands of its clients.

## Introduction

The prevalence of mental ill-health in the athletic population is a topic that has received increasing amounts of both media and research attention in recent years (cf. Hill et al., 2015; MacIntyre et al., 2015). Driven in part by the awareness-raising of elite athletes who have suffered and in some cases continue to suffer with mental ill-health, this topic is one that deserves the momentum it has attained. Amongst those who have spoken out about their struggles with mental ill-health are cricketers Jonathan Trott, Michael Yardy, Marcus Trescothick, and Matthew Hoggard; footballers Stan Collymore, Clarke Carlisle and Neil Lennon; cyclist Victoria Pendleton; track and field athlete, Dame Kelly Holmes; rugby union player Duncan Bell; boxer Frank Bruno and snooker player Ronnie O'Sullivan. Despite these attempts to put mental ill-health in sport under the spotlight, there remains a consensus that the true prevalence is under-reported.

Measuring the prevalence of mental ill-health in the athletic population is problematic. There are many barriers to reporting and seeking help for mental ill-health in this population, including (a) public, perceived, personal and self-stigmatizing attitudes to help-seeking, and (b) a lack of knowledge about mental health services on offer and the symptoms of mental disorders. Negative past experiences, lifestyle factors (e.g., a lack of time, money, or transportation) and personal characteristics such as gender are identified as personal obstacles to accessing assistance (Gulliver et al., 2012). Additionally, there are claims of sporting governing bodies attempting to downplay the significance of mental ill-health in the athletic population thereby raising concerns over the culture of these organizations (Reardon and Factor, 2010). Indeed, Bauman (2015) suggests that the culture of sport dictates that “mental toughness and mental health are seen as contradictory terms in the world of elite performance” (p. 1). These suggestions are reinforced by recent research commissioned by the Football World Players' Union, FIFPro, which confirmed that the reporting of mental ill-health in professional football is still considered taboo and therefore prevalence rates are likely to be vastly underestimated (Gouttebarge et al., 2015). There have, however been attempts to estimate the likely prevalence of mental ill-health in this population, with a number of studies concluding that it may in fact be on a level comparable with the general population (Yang et al., 2007; Markser, 2011; Bar and Markser, 2013). Bauman claims that this is unsurprising given “a growing number of complex and more intense mental health challenges” within this population, driven by the sporting environment (2015a, p. 1). Indeed, a number of studies have highlighted the role of the sport performance environment in increasing the risk of mental ill-health in athletes, focusing on issues such as: early sport-specialization, a loss of personal autonomy and disempowerment (Cresswell and Eklund, 2007), no opportunities to develop psychological coping skills (Bauman, 2015), sport-related stress (Noblet et al., 2002), living away from home (Bruner et al., 2008), limited social support due to relocation (Noblet and Gifford, 2002), disordered eating as a result of esthetic and weight-dependent sport (Sundgot-Borgen, 1994; Sundgot-Borgen and Torstveit, 2004), and high injury risk (Smith et al., 1990). To add further weight to this case, a recent literature review by Arnold and Fletcher (2012) identified that young elite athletes are faced with over 600 different stressors within their sport environment. These stressors pertain to a variety of matters including leadership, personal, team, cultural, environmental, and logistical issues. Arnold and Fletcher (2012) concluded that the existence of symptoms related to mental ill-health in this population is therefore unsurprising.

Notwithstanding the socio-contextual characteristics of competitive sport that may generate these mental health challenges, there is a further issue to consider: the sporting environment may exacerbate pre-existing mental ill-health as the full range of psychopathology is likely to exist within the athletic population (MacIntyre et al., 2015). Indeed, Bauman (2015) suggests that mental ill-health that began prior to involvement in sport may “become more evident when athletes are faced with stressors associated with elite sport” (p. 1). Either way, a continuous exposure to some, if not all of these challenges, has the potential to cause a deterioration in the athlete's well-being, carrying with it potentially negative outcomes such as common mental disorders (CMD) that may include anxiety and depression (Hughes and Leavey, 2012).

Common mental disorders (CMD) are defined as symptoms that relate to distress, anxiety, depression, substance abuse or dependence and are reported to be more frequent in young adults than in any other stage of life (Korten and Henderson, 2000; King et al., 2008; American Psychiatric Association, 2013). A recent study by Gouttebarge et al. (2015) was the first to examine the prevalence of CMD symptoms in current and former professional football (soccer) players across five European countries. They concluded that the prevalence of CMD ranged from 5% (burnout) to 26% (anxiety/depression) in 149 current players and from 16% (burnout) to 39% (anxiety/depression) in 104 former footballers. Gouttebarge et al. (2016) extended this research to retired rugby union players from France, Ireland and South Africa. The prevalence of CMD in this cohort ranged from 25% for distress, 28% for anxiety/depression, 29% for sleeping disturbance and 24% for adverse alcohol behavior. Elsewhere, there are suggestions that the athletic population as a whole are at *higher* risk of developing mental health problems such as eating disorders (Sundgot-Borgen and Torstveit, 2004), suicide (Baum, 2005), when experiencing “performance failure” (Rice et al., 2016, p. 12) and on retirement (Roberts et al., 2015; Gouttebarge et al., 2015). Others suggest prevalence rates of mental ill-health are comparable to the general population (Gulliver et al., 2015). Although, the details of the prevalence of CMD in the athlete population is imprecise, the aforementioned studies provide support for continuing to raise awareness of CMD within both populations, and elsewhere in sport, as a priority.

As awareness of CMD in sport is on the increase, so are the instances of practitioners encountering athletes presenting with problems related to these disorders. A small number of commentaries from those working in the sport and exercise science domain provide an insight to the issues faced. Firstly, observations by Morton and Roberts (2013) discussed the practitioners' (a nutritionist/physiologist and a sport psychology consultant) experiences of working with athletes suffering from the consequences of the relentless pursuit of success, fear of failure and balancing sport with other life commitments. Their article explained that athletes seeking assistance may approach the individual within their support team whom they feel most comfortable talking to, which is not always the most qualified or suitably trained person. Indeed, they make reference to the difficulties with athletes accepting referrals to other professionals (e.g., clinical psychologists) where there may be an absence of trust or skepticism surrounding the professionals' understanding of the challenges of the sporting environment. They finish by making a case for a review of the training of sport scientists to ensure that all practitioners develop an awareness of the likelihood of consulting with athletes experiencing CMDs, and to gain further skills (e.g., counseling) to help equip them adequately to deal with such a situation. Additional reference was made to the increased prevalence of athletes seeking sport psychology support for a combination of CMD and performance enhancement purposes in a conference presentation by Faull and Roberts (2014). Moreover, recent studies by Hill et al. (2015) and Newman et al. (2016) identified a wide range of mental health issues in young athletes involved in a sporting talent development environment and the negative impact of depression on sport performance, respectively. However, it was the dedication of a 60 minute panel discussion at the 30th Annual Conference of the Association for Applied Sport Psychology (AASP) in Indianapolis in 2015 that brought together sport psychology researchers and practitioners to examine the trend of CMD in the athletic population, concluding with a call to carry out further work to establish the nature and extent of the problem (MacIntyre et al., 2015).

Illustratively, given that one in four British adults will suffer with mental ill-health during the course of their lifetime (Mentalhealthorguk, 2016), and comparatively around 18% of the adult population in the United States of America (U.S.A.; Nihgov, 2016), combined with the suggestion that athletes are as (if not more) susceptible to mental ill-health as the general population, it is highly likely that practitioners in elite sport will encounter individuals suffering from CMD at some point in their career. Given that many athletes are often provided with a wide range of support services through their National Governing Bodies or clubs, or from private practice, there is an underlying debate regarding who is best placed to support athletes with mental health concerns. Anecdotally, there is a perception amongst personnel within the sporting domain that sport psychologists or sport psychology consultants are best placed to assist athletes in this predicament. For clarification, in the U.K. sport psychologists are licensed and accredited by the British Psychological Society (BPS) and the Health Care Professions Council (HCPC). Sport psychology consultants are usually sport scientists, accredited by the British Association of Sport and Exercise Sciences (BASES) as specialists in sport psychology. Further guidance on this distinction is featured later in this article.

Focusing on sport psychologists specifically, there is a difference in perspective between those who focus exclusively on performance issues, and those who add clinical issues as part of their service. The former group would refer any clinical issues to those trained to deal with them. Both groups are concerned with their clients welfare. One school of thought conceptualizes sport psychology as focusing exclusively on performance enhancement as opposed to clinical disorders (Hardy et al., 1996; Ravizza, 2001; Marchant and Gibbs, 2004). The other *balances* the athlete's performance with their welfare in a more holistic sense (Stambulova et al., 2006). Indeed, the holistic approach appears to support the long-held yet somewhat controversial concept that positive mental health increases the likelihood of success in sport (Morgan, 1985). This philosophical difference does not imply that performance focused practitioners are uncaring toward their clients, but rather their emphasis is on enhancing performance and letting other professionals take responsibility for mental health (for various reasons such as a lack of competence).

**Figure 1:**
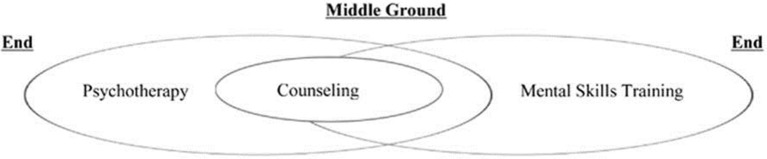
**The psychotherapy-mental skills training continuum**. Reproduced from Herzog and Hays (2012).

To further define the distinction in the services under debate, Herzog and Hays (2012) proposed a useful diagram illustrating the hypothetical continuum of psychotherapy to mental skills training in sport psychology consulting (see Figure 1).

To elaborate, psychotherapy is defined by Murphy and Murphy (2010) as “an intense process focused on helping clients deal with persistent and distressing life problems” (p. 13) and counseling as “the work of helping people cope with everyday problems and opportunities” (pp. 12–13). Mental skills training is considered to be an educational process that helps the client build particular skills in order to enhance performance (Steele, 2011). In the diagram above, counseling provides that middle ground between mental skills training and psychotherapy, which Herzog and Hays (2012) suggest may be imperceptible on a practical level. To further support these blurred lines in approaches to athlete support, in his recent book entitled *Being a Sport Psychologist*, Keegan (2016) suggests that sport psychology has “struggled to break free from the “mental toolkit” approach” (p. 49). He goes on to suggest that a well-rounded sport psychology service acknowledges a breadth of approach which may or may not encompass competing priorities between an athlete's performance and their well-being. Reflectively, he considers that focusing “purely on performance may severely constrain the effectiveness of the sport psychologist” (p. 50).

To demonstrate the reality of the practitioner's consulting approach to their clients, a range of case studies published by Herzog and Hays (2012) documented the complex “balance and shift” (p. 495) of psychotherapy and mental skills training in athletic consultations. Although, both authors are licensed mental health practitioners in the U.S.A., and thus may expect to encounter clients with mental health concerns, their cases presented highlight that the practitioner cannot always accurately predict the course that consultations will take, and that some will often require a change in tack. This mirrors the commentary by Morton and Roberts (2013) who discussed their experiences of working in high performance sport exposing them to situations where the distinction between performance-related and mental health concerns in athletes was somewhat blurred. To add weight to this argument, a large scale survey of sport psychology services provided to athletes at the U.S. Olympic Training Centre (USOTC) highlighted that in 85% of cases, sport psychology staff provided personal counseling to athletes (Meyers et al., 1995).

Given that sport psychology consultation may include the requirement for psychotherapy, counseling and mental skills training, an in-depth examination of the competencies required of sport psychologists and sport psychology consultants is necessary. The topic of competencies within this field is a complex one. Fletcher and Maher (2013) recently reviewed competency literature in professional psychology and its implications for applied sport psychology. They evaluated key documentation used in the training and development of sport psychologists such as the Association of Applied Sport Psychology (AASP) certification criteria (AASP, 2012), the International Society of Sport Psychology (ISSP) competencies position stand (Tenenbaum et al., 2003), the APA Proficiency in Sport Psychology checklist (Lesyk, 2005), and the first study to explore the competencies of applied sport psychologists by Ward et al. (2005). They concluded that the documentation available for the training and development of sport psychologists “does not adequately prepare trainees in all the necessary competencies” (p. 268), suggesting that there were six generic limitations, one of which was the lack of distinction between work focused on performance enhancement and therapeutic work with athletes (Aoyagi et al., 2012). This view mirrored the approach of Tod and Lavallee (2011) who had previously suggested that the “traditional” focus on performance enhancement and mental skills training was not adequate enough to meet the needs of the client in elite sport.

There are many lessons to be learned from the Herzog and Hays (2012) case studies, from the empirical evidence of the focus of sport psychology services at the USOTC (Meyers et al., 1995) to the review of competency literature by Fletcher and Maher (2013). However, each of the aforementioned articles relates to either sport psychology services in the USA or international competency standards for psychologists, which often vary between countries (Morris et al., 2003). Those variances have arisen from “diverse educational systems and philosophical differences among countries” (Gualberto Cremades et al., 2014, p. 3). In turn, this has led to great variation in the developmental patterns, certification, registration, licensure, accreditation, and process for the delivery of sport psychology services internationally. Accordingly, Gualberto Cremades et al. (2014) suggest the failure to address the requirement for a consistent set of competencies within the profession leads to “diverse methods of service delivery and training which may result in the blurring of the boundaries regarding what practice in the field is…” (p. 6).

To further illustrate the variance in approach to sport psychology provision internationally, the present article focuses on performance enhancement, CMDs and referral in the U.K. athletic population. The practice of sport psychology in the U.K. is nuanced (cf. McCarthy and Jones, 2013). First and foremost, the regulation of the psychology profession in the U.K. is governed by law. Practitioner psychologists are licensed and regulated through the Health and Care Professions Council (HCPC), a quasi-autonomous non-governmental organization (Quango). Training routes for sport psychologists typically involve a 3 year undergraduate degree accredited by the British Psychological Society (BPS), a BPS accredited master's degree in sport and exercise psychology, or the BPS qualification in sport and exercise psychology (QSEP) stage 1 and a further period of around 3 years of supervised practice through the QSEP stage 2 (British Psychological Society, 2014). This differs from clinical and counseling psychologists who typically undertake a 3 year professional doctorate at the end of their studies (e.g., McEwan and Tod, 2015). In the U.K. there is an alternative training route for those who do not wish to become practitioner psychologists. The British Association of Sport and Exercise Sciences (BASES) offers individuals accreditation as a sport and exercise scientist if they have completed a BASES endorsed undergraduate degree and a relevant MSc in a sport and exercise science-related discipline (e.g., sport and exercise psychology) and a further post-master's period of between 2 and 6 years of supervised experience. Through the BASES route, accredited sport and exercise scientists may work as mental skills coaches/trainers, sport performance consultants and sport psychology consultants. For further information on routes to practicing as a sport and exercise psychologist in the U.K. please see Cotterill (2011).

Sport psychology practitioners in the U.K. will often work in private practice, be employed directly by national governing bodies, individual sport teams or by a publically-funded quango such as the English Institute of Sport (EIS) or Sport Wales. Depending on the conditions of their appointment, they may be fully embedded in a high performance support team, contribute to it on a part-time or ad hoc basis, or they may work with individual athletes in isolation. In the U.K. the emphasis of sport psychology support is on performance enhancement. Indeed the training and supervision of sport psychologists or sport scientists in the U.K. involves minimal clinical psychology content, although elective courses aimed at continuing professional development (CPD) can help broaden skills. Service delivery models tend to be focused on psychological skills training and in some cases cognitive behavioral therapy (CBT) and humanistic counseling, depending on the practitioner's consulting philosophy. If, during the course of consultation with a client, practitioners are faced with an issue outside of their competency, the requirement is to refer the individual to the most appropriate professional for further assistance. Anecdotally, this situation arises on an increasingly regular basis in the U.K., where practitioners are faced with clinical issues that require that the individual be referred to a clinical psychologist.

In the U.K., a seemingly simple referral to a clinical psychologist has the potential to bring with it many complexities. If a client needs to see a clinical psychologist, they are often presented with two options: see a clinical psychologist through the National Health Service (NHS), or pay to see a clinical psychologist in private practice. In order to elaborate further, in the U.K., the majority of medical care is provided by the NHS which is free at the point of use. The NHS is a publically funded health care system for legal residents, paid for through general taxation. This is the option that individuals may pursue, especially if they are unable to meet the costs of private healthcare or if they do not hold private medical insurance. If the individual opts to see a clinical psychologist through the NHS, they may need a referral through a general practitioner (G.P.) or in some cases, depending on the facility, the individual may be able to self-refer. When seeking the assistance of a clinical psychologist in private practice, a referral can be arranged by the sport psychologist / sport psychology consultant or indeed a self-referral can be made. Regardless of their route to a clinical psychologist, the NHS will assign one to the individual on the basis of availability and geographical proximity. There are no guarantees that the clinical psychologist will have experience in working with the athletic population. Anecdotally, being referred to specialists with no knowledge of the sporting environment can lead to athletes being somewhat resistant to seeking such support for fear of not being understood. Additionally, the initial appointment with the clinical psychologist can take some time to occur, due to the length of waiting lists in the NHS. In some cases this can take up to 12 weeks.

In essence, it is the “blurring of the boundaries” of sport psychology practice that is the focus for this article. To summarize from the beginning: through the awareness-raising efforts of several high-profile current and former athletes, the issue of CMD in sport is gaining increasing attention, yet the prevalence is unclear and most likely, under-reported. Whilst the characteristics of the sporting environment may generate CMD within the athletic population, it also may exacerbate pre-existing conditions, and hence it is not surprising that sport psychology and sport science practitioners are anecdotally reporting increased incidences of athletes seeking support for CMDs. In a population where there are many barriers to reporting and seeking help for CMD, due in part to the culture of the high performance sporting environment, anecdotal reports suggest that those athletes asking for help are approaching personnel whom they are most comfortable talking to. In some cases, this may be a sport scientist or the sport psychologist or sport psychology consultant. Amongst personnel in the sporting domain, there is a perception that the sport psychologist or sport psychology consultant is best placed to assist athletes seeking assistance for CMD. However, sport psychology as a profession is split by two competing perspectives; one of which suggests that sport psychologists should work exclusively with athletes on performance enhancement, and another view that suggests adequately trained practitioners best serve their clients by being able to offer more than performance enhancement strategies. To add further complication, the development of the profession of sport psychology varies widely between countries, meaning that competence in this field is not always clearly defined.

In this article, the authors address these “blurred lines” in applied sport psychology by illustrating their experiences of practicing in the United Kingdom (U.K.). Two different case studies will be presented that highlight the complexities of supporting high performing athletes within the boundaries of the profession in the U.K. The first case study aims to demonstrate the dilemma that Practitioner A faced when the course of a consultation with an elite female lightweight rower took a deviation from the original scope of performance enhancement. The second case study focuses on Practitioner B's consultation with the medical team treating a professional rugby union player who had suffered a career-ending injury. The case involved Practitioner B assisting the medical team to “break bad news” to the player regarding the end of his professional rugby union career, before working with him to help him adhere to his rehabilitation programme and plan his transition out of the sport. The case details Practitioner B's suspicions that the athlete may have been suffering from depression and their attempt to refer him to a clinical psychologist for further assistance. In both instances, the data is presented in accordance with the recommendations of the University of Worcester's Ethics Committee with written informed consent from all subjects involved. All subjects gave written informed consent in accordance with the Declaration of Helsinki, including permission to disclose the details of their consultations. Any identifiers within the case studies have been removed to provide anonymity to the individuals and teams involved.

After the presentation of the two case studies, the article goes on to examine the professional guidelines that exist that govern the practice of sport psychology and an evaluation of their relevance to circumstances involving CMDs and referral encountered in applied practice. The article concludes with recommendations for the practice of applied sport psychology in order that it may continue to effectively meet the needs of client.

## Case study 1: Elite female lightweight rower

The athlete at the center of this case sought out the support of the sport psychology consultant (referred to herein as “Practitioner A”) through her rowing club, on relocation to the geographical area. Practitioner A had previously worked at several rowing clubs and had experience and contacts with selected coaches and athletes. The athlete was an elite lightweight rower and had been training for around 6 years when she sought assistance in handling pre-competition anxiety and “dealing with pressure.” After several weeks of support, building rapport and trust (e.g., Beckmann and Kellman, 2004; Fifer et al., 2008), the athlete disclosed a history of an eating disorder and explained how she felt that she was starting to “slip” and at “quite a rapid rate.” On hearing this information, Practitioner A reassured the athlete that this was useful information to know but made it clear that this was something that they may have to refer onto a clinical psychologist given their lack of clinical training.

The disclosure presented a challenge to Practitioner A for two reasons; firstly, a detailed intake session with the athlete had been conducted at the first meeting, where she had not disclosed any history of eating disorders despite being asked. Secondly, it was apparent that Practitioner A would now have to make decisions about how best to support the athlete given the nature of her disclosure which would involve the referral of the client to a clinical psychologist.

On examining the options for seeking the support of a clinical psychologist, the athlete explained how she was unable to meet the costs of a private practitioner. In addition, she did not hold private medical insurance; therefore, seeing a clinical psychologist through the NHS was the only option available to her. From previous experience, Practitioner A was aware that a referral of this nature could take up to 8 weeks. Having to potentially wait this long for an appointment with the clinical psychologist in a relatively urgent case such as this was considered detrimental to the athlete.

The further information raised a number of supplementary questions for Practitioner A to consider at this juncture; Firstly, how should the practitioner help maintain the well-being of the athlete until they were able to meet with the clinical psychologist? Secondly, how (if at all) should they continue with performance enhancement work in the interim period? The initial response from Practitioner A was to find out more about the eating disorder from the athlete, in an attempt to understand it better, to reassure her that other athletes have experienced similar issues, and to further understand how long she had experienced the feeling that she was “starting to slip.” She also wanted to understand the impact of the eating disorder on the athlete's rowing performance, what support she had been provided with in the past and the associated outcomes. Whilst all of these questions may appear logical, the paradox was that the practitioner was offering the athlete an opportunity to talk about her issues further, even though they knew that they were unable to directly assist her with the issue. In response, the athlete reported that the clinical psychologist whom she had seen previously was no longer working in the U.K. Furthermore, the athlete also reported that she would not choose to seek assistance from her in the future as she felt that the clinical psychologist lacked understanding of the demands of her particular sport.

In an attempt to expedite the process of clinical assistance to the athlete, Practitioner A requested details of the athlete's (G.P.) for the purposes of requesting a slightly different course of action in the form of a direct referral to a specialist in eating disorders from the local NHS. She was concerned that the athlete would not go to the G.P. of her own accord and that the clinical referral would take too long, so thought this course of action was the most supportive and expedient under the circumstances. The option was followed up and resulted in a 2 week wait for the athlete to see the specialist in eating disorders.

At this point in the process, Practitioner A considered whether she should continue to support the athlete while she was waiting for her first appointment with the eating disorder specialist, giving consideration to the consequences of reduced support in the interim. Furthermore, she evaluated the situation pertaining to the eating disorder. If this was the one central issue affecting performance, then the specialist in eating disorders would initially be in a position to intervene but the athlete may require a subsequent referral to a clinical psychologist. This was discussed with the athlete and they agreed that this would be the best course of action. In order to ensure the athlete was not made to feel abandoned, or their trust breeched, Practitioner A agreed to continue to support her with her performance-related issues. It was made clear that until the appointment date for the specialist for disordered eating came through, and indeed then the follow up clinical psychologist consultation, the practitioner would continue to be the primary point of contact.

In this case, the athlete began working with the specialist in disordered eating within 2 weeks; an initial appointment with the clinical psychologist took *an additional* 8 weeks, despite the athlete's health being comprised. Once the athlete had the first meeting with the specialist in disordered eating, the practitioner felt that she was able to pass the role of support onto this professional, on the proviso that the athlete would update the practitioner when the date finally came through for the clinical psychologist. When the consultation date with the clinical psychologist did eventually arrive, the athlete indicated that she felt the specialist knew nothing about sport, including the idiosyncrasies of lightweight rowing which made it extremely challenging for the athlete to feel understood. At this point, the athlete had established a good rapport with the specialist in disordered eating. The athlete therefore decided that she did not want to pursue the support with the clinical psychologist. Overall, the athlete felt she was provided with appropriate support and was offered the option to return to mental skills support with the practitioner in the future, should she decide it was what she needed or warranted once she had her eating disorder under control.

This case study serves to offer other practitioners with ideas about how to support athletes when the referral might not be the best fit. Similarly, the next case outlines the difficulty in the referral process in an injured professional male rugby union player.

## Case study 2: Injured professional rugby union player

The athlete at the center of this case study was a young professional rugby union player who had suffered a complete rupture of his right Achilles' tendon mid-season. The sport psychology consultant (“Practitioner B”) was initially contacted by the athlete's rugby club, for the purposes of providing advice to their medical team (Chief Medical Officer, Club General Practitioner, Chief Physiotherapist, Strength and Conditioning Coach and Lead Soft Tissue Therapist) regarding how to best communicate the diagnosis, prognosis and associated plans for rehabilitation to the injured individual. They explained that the player had, in their words, “over-identification” issues (e.g., Miller and Kerr, 2003). They felt that the injury and the associated recovery would present a great challenge to the athlete, hence requesting Practitioner B's support at this time. The medical team were recommending surgical repair of the tendon, which would, they said, involve around 6 months recovery. They were however, unable to provide certainty over the individual's future in the sport.

Practitioner B acknowledged that the medical team would be more familiar with the player, and as such would be able to best determine the most effective communication strategy. However, general advice on “breaking bad news” (e.g., Baile et al., 2000) was provided, which involves a six-step strategy for disclosing unfavorable information. With this strategy in mind, the practitioner advised the medical team to ensure that an open and honest dialog was maintained with the athlete at all times, whilst being mindful not to generate any false hope regarding a return to play at professional level. In addition, it was also recommended that the athlete be provided with as many opportunities as possible to exercise control over the situation (decision-making etc.) and the option of remaining as involved with the team as he wanted during his rehabilitation.

Regardless of the sensitive approach by the medical team, they reported that they felt the athlete had been unable to fully digest the news; they remained unconvinced that he fully understood the consequences of his injury. The medical team requested that Practitioner B meet with the player prior to his scheduled surgery to talk to him about his rehabilitation, and his future. The main objective, they explained, was to help the athlete come to terms with the severity of his injury and the impact it was likely to have on his future. Practitioner B did not know the athlete in question personally, but had a good working relationship with a number of the medical staff who she had worked with before in other sporting environments. A case conference was held where all details were discussed to ensure Practitioner B could gather background information before committing to the consultations.

The first encounter with the athlete was the day before his scheduled surgery. Practitioner B visited the rugby club, and found the athlete in the gym, engaged in upper-body resistance training. He explained that he was trying to maximize his chances of recovery and getting back into the squad. On further discussion, it was noted that he felt “scared,” not just about the prospect of surgery, he explained, but more the lack of certainty from the medical and coaching staff regarding his rugby playing future. He claimed that he was determined to prove everyone wrong, and that he would be returning to play in “no time.” The practitioner spent the consultation session with the athlete talking about the details of the surgery and the advice he had been given by his surgeon. Emphasis was placed on putting a goal setting strategy into place to focus his efforts on full recovery and rehabilitation in the forthcoming months.

During the second meeting, two weeks post-surgery, Practitioner B noted that the athlete's demeanor was “of concern.” She reported that he appeared non-communicative and withdrawn, explaining how receiving physiotherapy at the club every day was “driving him mad” (especially, he explained, when he saw his team mates training and carrying on as normal). He confirmed that he was in a great deal of pain, and was finding it difficult to get comfortable, was bored, highly emotional, and “just not interested in anything.” Having tried to review the individual's goals with him, and suggesting solutions such as: asking the physiotherapist to visit him at home until the training environment was less distressing for him, setting rehabilitation goals in conjunction with the medical team, arranging visits from his team mates to keep him busy and occupied. An agreement was made to return in 10 days or so to see how the athlete was progressing.

The third visit, 10 days later presented a contrasting experience. The individual was back at the club, and using the gym. As Practitioner B waited for him to come out of the fitness suite, the strength and conditioning coach approached and expressed his concern that the athlete was over-training, and not adhering to the rehabilitation programme that had been set. The athlete tried to convince Practitioner B that he had put his thoughts back in order, and explained he was “back on track.” He continued to state that he was determined to prove everyone wrong and that he would be back playing in “record time.” Given the open lines of communication agreed within the club setting, Practitioner B contacted the Chief Medical Officer to express the concerns over the individual's behavior and to gather further information from people who saw him on a day-to-day basis. This resulted in an agreement to hold a case conference the next day via conference call. During this discussion it became apparent that the player's recent behavior had been erratic, and his mood regularly and excessively fluctuating. When Practitioner B added this information to her own observations, she advised that a referral to a clinical psychologist should be considered, as there were signs and potential symptoms that required formal diagnosis and an appropriate intervention.

This suggestion was not met with enthusiasm by the club or the medical team. They recounted their previous experiences of clinical psychologists in a sporting environment, suggesting that they were often reluctant to visit the athlete and had little or no knowledge of the idiosyncrasies of the sporting environment, thereby impacting on their ability to be effective.

At the intake stage of the support, the medical team had indicated that Practitioner B's role would be sufficient to provide the support required and that their voice and opinion would be valued–yet the suggestion to involve a clinical psychologist was dismissed. Practitioner B therefore sought advice from peers (sport psychologists, sport psychology consultants, sports medicine specialists), looking for guidance and recommendations on how to continue to best support this athlete. Peers suggested some useful contacts that included clinical sport psychologists. Their details were gathered and cross checked with the HCPC. A short list of clinical psychologists and clinical sport psychologists was handed to the medical team with the recommendation that the athlete should be referred. The practitioner offered to attend the first consultations to help ease the transition from one practitioner to another.

The members of the medical team were not open to this recommendation, and insisted that Practitioner B continue to support the athlete. As Practitioner B felt uncomfortable leaving the individual without support, she continued to work with him to try to develop his self-awareness of his excessive rehabilitation efforts and how to best manage his non co-operation with the medical staff. During this time, the athlete expressed skepticism over the plans for referral and made clear that he did not want to engage in support from another individual.

The outcome of the consultations with the athlete proved unsuccessful in encouraging a change of behavior in the athlete, and ultimately, the excessive “rehabilitation” caused a re-rupture of his Achilles' tendon. Unfortunately, as predicted, the re-injury proved to be career-ending. On reflecting on this case, it is likely that the inability to refer the athlete to a clinical psychologist due to doubts over experience, and the ability to establish trust hindered the rehabilitation of the individual.

In summary, both cases presented highlight situations where a sport psychology consultant has encountered athletes presenting with CMDs that have been intertwined with a sport performance issue. Additionally, both cases illustrate barriers to seeking support from further specialists such as clinical psychologists. The first case summarized the time delay and potential lottery associated with engaging support via the NHS, and the second, the negative perception of external specialists held by athletes and their support personnel. In both cases, there was an underlying assumption that the sport psychology consultant was best placed to help both athletes with their CMDs. Using these cases as examples, it is suggested that the inability of sport psychology consultants in the U.K. to assist athletes with such common disorders requires further reflection. To further understand how this practice may be developed, the next section examines the practice of sport psychology in the U.K. against the backdrop of the guidance and regulation of sport psychologists and sport psychology consultants globally.

## Global perspectives on sport psychology training and regulation

For advice and guidelines on how best to support athletes presenting a wide variety of problems such as CMDs, the natural place to start is with relevant professional bodies. Given the globalization of the profession, a universal view is needed to appreciate the context in which practitioners are trained and working in the field. Whilst globalization is a testament to the strength of the field, the practice of sport psychology differs from country to country and even between different states in the U.S.A.

Starting in the U.S.A., the primary professional body supporting applied sport psychologists is the Association of Applied Sport Psychology (AASP). AASP runs an online support system for continuous educational development including webinars and a platform for sharing resources (see http://www.appliedsportpsych.org/certified-consultants/ for further details). In addition, AASP resources include an annual conference, an established certified consultant's programme and opportunities for student and professional development. AASP is the only professional body to detail what is considered to be *outside of the scope* of the service provided by a certified consultant (sport psychology practitioner). This is communicated clearly in their Internship and Practicum Experience Database Manual (IPED; AASP, 2013), which states that the following activities are exempt: “diagnosis or treatment of psychopathology, treatment of substance abuse disorders (including alcoholism and other types of chemical dependencies), eating disorders, obesity, and any marital and family therapy.” The IPED (AASP, 2013) provides useful guidance for practitioners in delineating their role by stating that if an appropriate referral is not made when consulting with a client with such issues, the practitioner's behavior may be deemed to be unethical.

Applied sport psychology training and education is continuously evolving in the face of more common instances of athletes seeking assistance for issues beyond the scope of performance psychology (Portenga et al., 2011). With opportunities available to seek additional training to “up skill” and advance understanding and knowledge in some of the aforementioned areas, we are faced with a further blurring of the lines regarding when a referral should take place (cf. Herzog and Hays, 2012). Indeed, Herzog and Hays (2012) make it clear that it is the individual practitioner's training that drives the nature of the support to the athlete, and the point at which a referral should be made (if at all). Although, the guidelines are clear, there may still be instances in which practitioners vary in terms of their competencies, nevertheless they need to be aware of when to make referrals given the nature of their expertise. Ultimately, it is the self-awareness and integrity of the practitioner to know their limits and competency level based on their training that is relied upon.

The European Federation of Sport Psychology (FEPSAC) has issued a position statement on ethics (Fepsac, 2011) and an accompanying ethical checklist (Little et al., 2011), the detail of which puts the onus on the practitioner to judge whether they are practicing within their qualifications, expertise and experience. If they conclude that they are unable to assist their client with a specific problem, a referral is recommended. The mechanics of the referral process however, remain somewhat elusive. Australia uses a slightly different approach. In a recent article by Wensley (2013) the Australian Institute of Sport (AIS) raises the question of whether it is appropriate for a sport psychologist to help an athlete with a mental health problem. Wensley (2013) states that in Australia, sport psychologists are trained to work with people with the most common mental health problems, including depression, and anxiety. However, she suggests that they may choose to refer the athlete if the conclusion was that they would be better served by a mental health specialist. Regardless of this slight difference of approach, no guidelines for referral practices were evident.

In the U.K., BASES, and the Division of Sport and Exercise Psychology (DSEP) of the BPS work together to promote “excellence in research, teaching and practical applications in sport and exercise psychology” (British Association of Sport and Exercise Sciences (BASES), 2014). Both organizations “share the common goals of ensuring that individuals, teams and organizations receive best practice in the provision of psychological services in sport and exercise settings” (British Association of Sport and Exercise Sciences (BASES), 2014). Certainly, the BASES Code of Conduct (British Association of Sport and Exercise Sciences (BASES), 2009) helps members understand that they must work within their competency levels in terms of their “qualifications, experience and expertise” (p. 2) requiring that any matter that lies within other areas of specialism such as medically-related issues or those associated with the role of a physiotherapist should be “referred to an appropriate professional within such a field” (p. 2). The BPS Code of Ethics and Conduct (British Psychological Society, 2009) similarly states that psychologists are required to “refer clients to alternative sources of assistance as appropriate, facilitating the transfer and continuity of care through reasonable collaboration with other professionals” (p. 19). However, despite their cited objectives and the clear conduct requirements of each, both still appear to fall short on providing specific and easily accessible guidelines beyond recognizing the situations when a referral may be needed. To elaborate, those working in the field of applied sport psychology are bound by their respective accrediting bodies (e.g., British Psychological Society; BPS, or state licensing boards in the U.S.A.) which in summary require practitioners to work within the boundaries of their competence (British Psychological Society, 2009). However, the process of how to support the athlete during the referral process is rarely discussed and competency, as highlighted by Fletcher and Maher (2013), is a complex issue.

Having considered some of the challenges of ensuring that the practice of applied sport psychology continues to meet the needs of the client and concluding that there is a lack of specific guidelines, recommendations follow that may help guide practitioners when encountering athletes contending with CMDs during the course of consultation.

## Referral

Most requests for performance enhancement assistance for athletes are likely to be what they appear to be, and suitably qualified practitioners will be able to help. Sometimes, however, as illustrated above, athletes present with issues outside practitioners' realms of expertise, or concerns may emerge after practitioners and athletes have worked together for a period of time. Referral is suitable in these cases. The possibility of referral is relevant to both trainees and experienced practitioners. Given the wide range of mental disorders that exist, and the various ways in which athletes' lives can be disrupted, even seasoned practitioners may find that they are ill-equipped to assist on occasion.

### Considering whether to refer

In considering whether referral is a suitable option, Tod and Andersen (2015) presented guiding questions to help practitioners decide. For example: How long has the issue existed? What is the severity of the issue? What role does the issue play in the person's life? Are there displays of unusual emotions or behaviors around the issue? How well are the athlete's existing coping strategies developed? Does the practitioner have the competencies, knowledge, skills, and experience to address the issue? Issues that are recent, not severe in their emotional implications, and do not have substantial overlap with other aspects of a person's life are less likely to require referral. Unusual emotional reactions that are out of character, or out of place, may also warrant consideration for referral. For example, an athlete who is facing a tough competition and who experiences mild to moderate anxiety and negative self-talk is not likely to require referral. A person for whom each athletic competition is an all-or-nothing battle for self-identity, whose emotional state is dependent on performance outcomes, and where strong anxiety, depressive states, or substance abuse may also be involved, is more likely to need a referral. In such cases, however, performance or sport psychology practitioners can still address performance-related issues.

### Raising the issue of referral

There may be times when sport psychologists are uncertain how athletes will react to referral suggestions or may be unsure of the best way to raise this advice with a particular client. In these situations, seeking counsel from mentors, supervisors, or colleagues may help ease the burden and provide constructive direction. Practitioners could also engage in role plays to prepare themselves. As well as exploring ways that might facilitate referral, role plays can also give practitioners insights into how they might react to athlete resistance or exuberant enthusiasm. In addition to seeking advice from colleagues and practicing potential scenarios, documenting reasons for suggesting referral, the interaction, athletes' responses, and outcomes, as part of the practitioner's case notes, will form a foundation for future planning and decision making. It may also help protect practitioners from negative consequences if they can demonstrate they had made suitable attempts, and had observed ethical and legal requirements, to help clients access the assistance they needed.

### Barriers to referral

The stigma of seeking psychological assistance is alive and well, and well-documented, especially in the sporting domain (Clement et al., 2015). Indeed this stigma may be inadvertently propagated by overzealous sport psychologists, eager to promote concepts such as mental toughness. Additionally, as the second case study highlighted, athletes and their support staff may not act on practitioners' suggestions to seek help from another professional for any of several reasons (Van Raalte and Andersen, 2013). For example, in the absence of close relationships, athletes may not trust that the practitioners have their best interests at heart. Practitioners' recommendations might be interpreted as attempts to rid themselves of their clients. If handled insensitively, athletes might feel unsupported and believe their anxieties regarding referral have been ignored. One fear might be that the mental health practitioners will take away from athletes what made them high performers. Perceived threats to confidentiality may influence athletes' actions. They may fear that if word gets around they are seeing other practitioners, they might feel stripped of their dignity. Practitioners may not prepare athletes adequately for the referral. Sport psychologists and sport psychology consultants need to inform athletes about what is involved, whom the other helpers include, why they might help, and the implications for the existing sport psychologist—athlete relationships. Practitioners can signal to athletes in their first sessions together that referral might be a suggestion raised in the future. Athletes poorly prepared may have unrealistic or inaccurate expectations about the new practitioner. In the absence of follow-up or facilitation, athletes or their support staff might not contact the recommended practitioners or persist after initial meetings.

### Referring in

Often, referral procedures are not straightforward for many reasons, as illustrated in the case studies presented above. If trust has been built between sport psychologists and athletes, sending clients directly to someone else when issues beyond practitioners' competencies arises may not be optimal. “*Referring in”* may be the better choice (Van Raalte and Andersen, 2013). Bringing in a qualified professional and having the three parties discuss and plan support together may be less threatening to the athlete and at the same time, ease the individual into a relationship with the new helping professional. Referring in implies that practitioners have adequate networks that include various types of helping professionals whom they trust. These networks might include helping professionals such as clinical psychologists, counselors, psychiatrists, sport scientists, and nutritionists.

### When referral is unsuccessful

The match between the athlete and the helper may not be sufficient for benefits to accrue. It may have been a huge step for athletes to share sensitive material with sport psychology practitioners, who may be among the few trusted people they feel able to confide in. When faced with referrals that do not appear to be working well, sport psychologists can still keep in contact with athletes. Avoiding the perception that the sport psychology practitioner's continued help is conditional on the athlete meeting with the external helper will help maintain a close relationship. It is inadvisable and impractical to force athletes to meet with other professionals, except in situations where there is a threat of harm to self or others (where there are then ethical and legal obligations to uphold). Sport psychologists can continue to provide performance enhancement assistance and can initiate the referral process in the future if athletes change their minds.

## Professional development activities

### Education and training

Given the evidence presented in the current manuscript, it is realistic that practitioners will come across athletes displaying the signs and symptoms of the CMDs that the general population also experience. Regarding the education of current and future trainees, professional bodies, such as the British Psychological Society and education providers could help students prepare for their careers and provide clients with high quality services by ensuring that information about CMDs and the skills needed to provide a minimum level of help in such cases are included in educational pathways. Currently, for example, the BPS training documentation (both stage 1 and 2) does not treat learning about specific CMD as core. In a recent systematic review, Bratland-Sanda and Sundgot-Borgen (2013) noted that the rates at which athletes experience eating disorders were considerably higher than compared to the general population. Therefore, there is a strong justification for such content to be made integral to training pathways.

The definition of the phrase above “provide a minimum level of help” will vary. All practicing sport psychologists and sport psychology consultants might be expected to be able to (a) identify the signs and symptoms of CMDs, (b) talk to clients about their observations, and (c) help athletes obtain the assistance they need to cope with or resolve their issues. Some practicing sport psychologists may have the training and experience needed to assist directly, whereas others may need to implement referral procedures. Given the difficulties and relationship strains that can arise during referral, sport psychology practitioners who have the skills to assist clients with CMDs, may be better placed to provide that support.

### Develop relevant knowledge and skills

Consultants can prepare themselves for helping clients with CMDs by engaging in learning activities allowing them to develop the necessary skills and knowledge to be able to help these athletes in some way. Such learning activities could include reading a variety of sources, including research, theory, diagnostic and treatment manuals, and biographical accounts of people living with issues such as depression, eating disorders, and anxiety, along with others associated with sport and exercise contexts. The American Psychiatric Association's (2013) Diagnostic and Statistical Manual-5 (DSM-V) is useful for gaining an overview of diagnosable mental health disorders. Reading the DSM-V, however, would be insufficient on its own to prepare practitioners to help clients. Such reading may be supplemented with supervision and training in current research, theory, and practice on helping people (see below). Practitioners will also benefit from realizing that clients who are not displaying signs and symptoms sufficient to warrant a formal diagnosis may have sub-clinical levels of disorders for which they need help. In addition, athletes may have mental health or emotional concerns that fall outside of diagnosable problems. Examples include identity issues, sexual orientation and abusive environments, sexual health issues, alcohol, drug and substance use, anger and aggression control, romantic and family involvement, and abuse of power in the sporting context (Tod and Andersen, 2015).

To supplement empirical and theoretical literature, biographical accounts and case studies of individuals experiencing mental ill-health may help practitioners appreciate what life is like for these individuals and the social contexts they may live within. In recent years, a number of high profile, and not so recognizable, athletes (and other performers) have published their stories, discussing their experiences with a range of issues including eating disorders, depression, substance abuse, and discrimination (e.g., Fussell, 1991; Bruno and Mitchell, 2006; Trescothick, 2009; de Rossi, 2011). Practitioners might also consider recommending these often fascinating and moving accounts to their clients if they believe the individuals will benefit. Example benefits might include finding comfort in learning how other people have coped, that they are not unique, and that there can be reasons for hope. Also, clients might gain practical ideas and strategies for coping and resolving issues.

In addition to expanding their knowledge base, practitioners can develop the skills to interact with and help clients. Relationship building, communication, and counseling skills represent one set of abilities, and as examples of the common factors in service delivery, may be differentiated from the specific factors associated with interventions (Wampold and Budge, 2012). If referral is the suitable course of action, solid counseling skills will likely help practitioners assist clients to receive the desirable help they need. If practitioners are finding that they are referring clients for similar issues, for example, disordered eating, then they might consider developing the skills to help these individuals themselves (within the legal and ethical constraints allowed in their region of practice). Referral procedures are not always seamless, as illustrated in the current manuscript for systemic, logistical and interpersonal reasons. Performance-focused practitioners with the skills to help people with CMD and who can navigate the ethical and other issues that accompany a shift in service delivery focus may assist clients more than relying on referral procedures.

### Gain supervised experience

Although, building knowledge and skills contributes, competence also results from actual experience in helping clients with specific issues. Such interactions provide practitioners with insights into the helping process that are difficult to learn from traditional classroom based teaching methods. For example, actually supporting a person experiencing depressed mood helps practitioner become aware of how they react to such individuals and how their own reactions might be helpful or not to their clients.

The tension that arises with developing experience is that there is always a “first client” with whom practitioners have to “cut their teeth.” Such an observation is not limited to trainees, and even experienced practitioners may come across new issues for which they are not trained. In such instances client health and well-being have priority over practitioner professional development, and the challenge is for psychologists to find suitable and legal ways to develop their toolbox. Formal supervised experience represents an avenue that could be explored, with the selection of a suitable supervisor (i.e., somebody with the necessary qualifications, professional body approval, and experience with the issue).

### Engage in lifelong professional reconfiguration and evolution

It is a cliché to suggest that practitioners need to engage in lifelong learning and continual professional development, and it may not best encapsulate the key points involved. Perhaps the phrase “practitioners need to engage in lifelong professional reconfiguration and evolution” might communicate the sentiments more clearly. Evolution involves the notion that species continually adapt to fit in their current niches and environments. In a sense, species reconfigure their attributes as a way to ensure survival. Parallels exist for applied sport psychologists and sport psychology consultants. Both society at large, and the sporting world in particular, are dynamic and in a constant state of flux. For example, environments change, new technologies are implemented, the ways people interact broadens, and athletes experience new issues or old issues in different ways. Over time, practitioners' skills and knowledge may become less effective or even outdated. Jennings and Skovholt (1999) suggested that a voracious desire to learn was an attribute of master therapists, and such a commitment to professional reconfiguration will assist in practitioners maintaining an adequate “fit” for the clients they hope to serve and environments in which they wish to operate.

## Conclusion

In conclusion, elite athletes exist within an environment that may generate, or indeed exacerbate CMDs due the unique range of stressors involved. Yet, it is believed that the prevalence of CMDs in elite sport is under-reported due to a number of barriers that include stigma and a lack of awareness. However, there are increased reports of sport psychologists, sport psychology consultants and other practitioners in elite sport reporting athletes seeking support for CMDs, often at the same time as seeking performance enhancement assistance. Paradoxically, in certain circumstances, the sport psychologist or sport psychology consultant is not always the best individual to provide the support, given the blurred lines that exist in the practice of applied sport psychology. The management of the mental health needs of elite athletes often require referral to a clinical psychologist; however, this person may lack context-specific knowledge of the sporting environment which impacts negatively on their ability to provide effective intervention. In order that athletes with CMDs can continue to be appropriately supported, a number of recommendations for sport psychologists and sport psychology consultants are made. These include suggestions for effective referral procedures, the call for a review of the education and training of sport psychologists to put CMDs in sport at the core of professional training, the continual development of knowledge and skills related to CMDs (e.g., counseling and communication), and the recommendation that those working in the field of applied sport psychology engage in a process of lifelong professional reconfiguration and evolution for the purposes of ensuring that they are able to respond to the demands of their clients.

## Author contributions

CMR co-ordinated the writing of this manuscript, compiled the introduction, second case study and the conclusion, edited the manuscript and acts as the corresponding author. AF added the first case study and the section on Global Perspectives. DT added the recommendations section.

### Conflict of interest statement

The authors declare that the research was conducted in the absence of any commercial or financial relationships that could be construed as a potential conflict of interest. The reviewer BG and handling Editor declared their shared affiliation, and the handling Editor states that the process nevertheless met the standards of a fair and objective review.
